# A New Expanded Mixed Element Method for Convection-Dominated Sobolev Equation

**DOI:** 10.1155/2014/297825

**Published:** 2014-02-18

**Authors:** Jinfeng Wang, Yang Liu, Hong Li, Zhichao Fang

**Affiliations:** ^1^School of Statistics and Mathematics, Inner Mongolia University of Finance and Economics, Hohhot 010070, China; ^2^School of Mathematical Sciences, Inner Mongolia University, Hohhot 010021, China

## Abstract

We propose and analyze a new expanded mixed element method, whose gradient belongs to the simple square integrable space instead of the classical *H*(div; Ω) space of Chen's expanded mixed element method. We study the new expanded mixed element method for convection-dominated Sobolev equation, prove the existence and uniqueness for finite element solution, and introduce a new expanded mixed projection. We derive the optimal a priori error estimates in *L* ^2^-norm for the scalar unknown *u* and a priori error estimates in (*L* ^2^)^2^-norm for its gradient **λ** and its flux **σ**. Moreover, we obtain the optimal a priori error estimates in *H* ^1^-norm for the scalar unknown *u*. Finally, we obtained some numerical results to illustrate efficiency of the new method.

## 1. Introduction

We consider the following Sobolev equation with convection term:

(1) ut+c(x)·∇u−∇·(a(x,t)∇u+b(x,t)∇ut)=f(x,t),(x,t)∈Ω×J,u(x,t)=0, (x,t)∈∂Ω×J¯,u(x,0)=u0(x), x∈Ω,

where *Ω* is a bounded convex polygonal domain in *R* ^2^ with *Lipschitz *continuous boundary ∂*Ω* and *J* = (0, *T*] is the time interval with 0 < *T* < *∞*. *u*_0_(**x**) and *f*(**x**, *t*) are given functions, coefficients *a* = *a*(**x**, *t*), *b* = *b*(**x**, *t*) are smooth and bounded functions, coefficient **c**(**x**) = (*c*_1_(**x**), *c*_2_(**x**)) is a bounded vector, and

(2) $$\begin{array}{c} A_{1}:0<a_{0}\leq a\left(x,t\right)\leq a_{1}<+\infty ;\\ A_{2}:0<b_{0}\leq b\left(x,t\right)\leq b_{1}<+\infty ,\;\left|b_{t}\left(x,t\right)\right|\leq d_{1}<+\infty ;\\ A_{3}:0<{\left(\sum_{i=1}^{2}c_{i}^{2}(x)\right)}^{1/2}<+\infty , \end{array}$$

for some positive constants *a*_0_, *a*_1_, *b*_0_, *b*_1_, and *d*_1_.

Sobolev equations are a class of important evolution partial differential equations and have a lot of applications in many physical problems, such as the porous theories concerned with percolation into rocks with cracks, the heat conduction problems in different mediums, and the transport problems of humidity in soil. In [1], the finite element method for nonlinear Sobolev equation with nonlinear boundary conditions was studied. In [2], a discontinuous Galerkin method for Sobolev equation was studied. In [3–7], some mixed finite element methods for Sobolev equations are studied and analyzed.

In 1994, Chen [8, 9] developed and studied an expanded mixed element method and proved some mathematical theories for second-order linear elliptic equation. Compared to standard mixed element methods the expanded mixed method is expanded in the sense that three variables are explicitly approximated, namely, the scalar unknown, its gradient, and its flux. From then on, the expanded mixed element method has been applied to solving other partial differential equations [10]. At the same time, many researchers proposed and studied some new numerical methods based on Chen's expanded mixed method, such as expanded mixed hybrid methods [11], two-grid expanded mixed finite element method [12–14], expanded characteristic-mixed element method [15], expanded mixed covolume method [16, 17], and expanded positive definite mixed method [18].

In 2011, we developed and analyzed a new expanded mixed finite element method [19] for elliptic equations based on the mixed schemes [20, 21] which have been studied for some partial differential equations [4, 22–25]. Compared to Chen's expanded mixed method, the gradient for the new expanded mixed method belongs to the simple square integrable space instead of the classical *H*(div⁡; *Ω*) space. In this paper, we will study the new expanded mixed element method for convection-dominated Sobolev equation. We will give the proof for the existence and uniqueness of the solution for semidiscrete scheme and a new expanded mixed projection and the proof of its uniqueness. We will prove the optimal a priori error estimates in *L* ^2^-norm for the scalar unknown *u* and a priori error estimates in (*L* ^2^)^2^-norm for its gradient *λ* and its flux *σ*. In particular, we obtained the optimal a priori error estimates in *H* ^1^-norm for the scalar unknown *u*. Finally, we obtained some numerical results to confirm our theoretical analysis.

Throughout this paper, *C* will denote a generic positive constant which is free of the space-time parameters *h* and Δ*t*. At the same time, we denote the natural inner product in *L* ^2^(*Ω*) or (*L* ^2^(*Ω*))^2^ by (·, ·) with the corresponding norm ||·||. The other notations and definitions of Sobolev spaces as in [26] are used.

## 2. New Expanded Mixed Formulation

Introducing the two auxiliary variables *λ* = ∇*u*, *σ* = −(*a*(**x**, *t*)∇*u* + *b*(**x**, *t*)∇*u*_*t*_) = −(*a*(**x**, *t*)*λ* + *b*(**x**, *t*)*λ*_*t*_), we obtain the following first-order system for (1):

(3) $$\begin{array}{c} u_{t}+c\cdot \lambda +\nabla \cdot \sigma =f\left(x,t\right),\\ \lambda -\nabla u=0,\\ \sigma +\left(a\lambda +b\lambda_{t}\right)=0. \end{array}$$

Using Chen's expanded mixed method, the mixed weak formulation for problem (1) is to find {*u*, *λ*, *σ*}:[0, *T*] ↦ *W* × **V** × **X** such that

(4) (a) (ut,v)+(c·λ,v)+(∇·σ,v)=(f,v), ∀v∈W,(b) (λ,w)+(u,∇·w)=0, ∀w∈V,(c) (σ,z)+(aλ+bλt,z)=0, ∀z∈X,

where *W* = *L* ^2^(*Ω*) or *W* = {*w* ∈ *L* ^2^(*Ω*) | *w*|_∂*Ω*_ = 0}, **V** = **H**(div⁡; *Ω*) = {**v** ∈ (*L* ^2^(*Ω*))^2^ | ∇·**v** ∈ *L* ^2^(*Ω*)}, and **X** = (*L* ^2^(*Ω*))^2^.

In this paper, the new expanded mixed weak formulation of (3) is to find {*u*, *λ*, *σ*}:[0, *T*] ↦ *H*_0_^1^ × (*L* ^2^(*Ω*))^2^ × (*L* ^2^(*Ω*))^2^ such that

(5) (a) (ut,v)+(c·λ,v)−(σ,∇v)=(f,v), ∀v∈H01,(b) (λ,w)−(∇u,w)=0, ∀w∈(L2(Ω))2,(c) (σ,z)+(aλ+bλt,z)=0, ∀z∈(L2(Ω))2.

Then, the semidiscrete mixed finite element scheme for (5) is to determine {*u*_*h*_, *λ*_*h*_, *σ*_*h*_}:[0, *T*] ↦ *V*_*h*_ × **W**_*h*_ × **W**_*h*_ such that

(6) (a) (uht,vh)+(c·λh,vh)−(σh,∇vh)=(f,vh), ∀vh∈Vh,(b) (λh,wh)−(∇uh,wh)=0, ∀wh∈Wh,(c) (σh,zh)+(aλh+bλht,zh)=0, ∀zh∈Wh,

where (*V*_*h*_, **W**_*h*_) is chosen as the finite element pair *P*_1_ − *P*_0_^2^ as follows:

(7) Vh={vh∈C0(Ω)∩H01 ∣ vh∈P1(K),∀K∈𝒦h},Wh={wh=(w1h,w2h)∈(L2(Ω))2 ∣ w1h,w2h∈P0(K),∀K∈𝒦h}.

From [20, 21], we find that (*V*_*h*_, **W**_*h*_) satisfies the so-called discrete Ladyzhenskaya-Babuska-Brezzi condition.

**Remark 1**

Compared to Chen's expanded mixed weak formulation (4), the gradient in the scheme (5) belongs to the simple square integrable space (*L* ^2^(*Ω*))^2^ instead of the classical **H**(div⁡; *Ω*) space. Obviously, the regularity requirements on the solution *λ* = ∇*u* reduced.

**Theorem 2**

There exists a unique discrete solution to semidiscrete scheme (6).

**Proof**

Let {*ψ*_*i*_(**x**)}_*i*=1_^*n*_1_^ and {*φ*_*j*_(**x**)}_*j*=1_^*n*_2_^ be bases of *V*_*h*_ and **W**_*h*_, respectively. Let

(8) $$\begin{array}{c} u_{h}=\sum_{i=1}^{n_{1}}u_{i}\left(t\right)\psi_{i}\left(x\right),\;\;\lambda_{h}=\sum_{j=1}^{n_{2}}\lambda_{j}\left(t\right)\varphi_{j}\left(x\right),\\ \sigma_{h}=\sum_{k=1}^{n_{2}}\sigma_{k}\left(t\right)\varphi_{k}\left(x\right),\;\;u_{h}\left(0\right)=\sum_{i=1}^{n_{1}}{\bar{u}}_{i}\psi_{i}\left(x\right),\\ \lambda_{h}\left(0\right)=\sum_{j=1}^{n_{2}}{\bar{\lambda }}_{j}\varphi_{j}\left(x\right),\;\;\sigma_{h}\left(0\right)=\sum_{k=1}^{n_{2}}{\bar{\sigma }}_{k}\varphi_{k}\left(x\right), \end{array}$$

and substituting these expressions into (6) and choosing *v*_*h*_ = *ψ*_*m*_, **w**_*h*_ = *φ*_*l*_, and **z**_*h*_ = *φ*_*s*_, the problems (6) can be written as follows: find $\left\{\tilde{u}(t),\tilde{\lambda }(t),\tilde{\sigma }(t)\right\}$ such that, for all, *t* ∈ (0, *T*]

(9) (a) Au~′(t)+Bλ~(t)−Cσ~(t)=F(t),(b) Dλ~(t)−Eu~(t)=0,(c) Dσ~(t)+Hλ~(t)+Jλ~′(t)=0,(d) u~(0)=u¯,  λ~(0)=λ¯,  σ~(0)=σ¯,

where

(10) $$\begin{array}{c} A={\left(\left(\psi_{i},\psi_{m}\right)\right)}_{n_{1}\times n_{1}},\;\;B={\left(\left(c\cdot \varphi_{j},\psi_{m}\right)\right)}_{n_{1}\times n_{2}},\\ C={\left(\left(\varphi_{s},\nabla \psi_{m}\right)\right)}_{n_{1}\times n_{2}},\;\;D={\left(\left(\varphi_{j},\varphi_{l}\right)\right)}_{n_{2}\times n_{2}},\\ E={\left(\left(\nabla \psi_{i},\varphi_{l}\right)\right)}_{n_{2}\times n_{1}},\\ H={\left(\left(a\varphi_{j},\varphi_{s}\right)\right)}_{n_{2}\times n_{2}},\;\;J={\left(\left(b\varphi_{j},\varphi_{s}\right)\right)}_{n_{2}\times n_{2}},\\ \tilde{u}\left(t\right)={\left(u_{1}\left(t\right),u_{2}\left(t\right),\ldots ,u_{n_{1}}\left(t\right)\right)}^{T},\\ \tilde{\lambda }\left(t\right)={\left(\lambda_{1}(t),\lambda_{2}(t),\ldots ,\lambda_{n_{2}}(t)\right)}^{T},\\ \tilde{\sigma }\left(t\right)={\left(\sigma_{1}(t),\sigma_{2}(t),\ldots ,\sigma_{n_{2}}(t)\right)}^{T},\\ F\left(t\right)={\left(\left(f,\psi_{m}\right)\right)}_{n_{1}\times 1},\;\;\bar{u}={\left({\bar{u}}_{1},{\bar{u}}_{2},\ldots ,{\bar{u}}_{n_{1}}\right)}^{T},\\ \bar{\lambda }={\left({\bar{\lambda }}_{1},{\bar{\lambda }}_{2},\ldots ,{\bar{\lambda }}_{n_{2}}\right)}^{T},\;\;\bar{\sigma }={\left({\bar{\sigma }}_{1},{\bar{\sigma }}_{2},\ldots ,{\bar{\sigma }}_{n_{2}}\right)}^{T}. \end{array}$$

It is easy to see that **A** and **D** are invertible matrixes. From (9), the initial value problems can be written as follows:

(11) (a) u~′(t)+A−1Bλ~(t)−A−1Cσ~(t)=A−1F(t),(b) λ~(t)=D−1Eu~(t),(c) σ~(t)+D−1Hλ~(t)+D−1Jλ~′(t)=0,(d) u~(0)=u¯,  λ~(0)=λ¯,  σ~(0)=σ¯.

Substitute (11)(b) into (11)(c) to get

(12) $$\begin{array}{c} \tilde{\sigma }\left(t\right)=-D^{-1}HD^{-1}E\tilde{u}\left(t\right)-D^{-1}JD^{-1}{E\tilde{u}}^{\prime }\left(t\right). \end{array}$$

Substituting (11)(b) and (12) into (11)(a), we obtain

(13) (I+A−1CD−1JD−1E)u~′(t) +A−1(B+CD−1H)D−1Eu~(t)=A−1F(t),

where **I** is a unit matrix.

Thus, by the theory of differential equations [27], (13) has a unique solution $\tilde{u}(t)$; then (12) and (11)(b) have unique solutions $\tilde{\sigma }(t)$ and $\tilde{\lambda }(t)$, respectively. Equivalently (6) has a unique solution.

## 3. Error Estimates for Semidiscrete Scheme

In order to analyze the convergence of the method, we first introduce the new expanded mixed elliptic projection associated with our equations.

Let (*u*_*h*_, *λ*_*h*_, *σ*_*h*_):[0, *T*] → *V*_*h*_ × **W**_*h*_ × **W**_*h*_ be given by the following mixed relations:

(14) (a) (σ−σ~h,∇vh)+(c·(λ−λ~h),vh)=0, ∀vh∈Vh,(b) (λ−λ~h,wh)−(∇(u−u~h),wh)=0, ∀wh∈Wh,(c) (σ−σ~h,zh)+(a(λ−λ~h)+b(λt−λ~ht),zh)=0,∀zh∈Wh.

**Theorem 3**

There exists a unique solution to the new expanded mixed elliptic projection (14).

**Proof**

Noting that mixed elliptic projection system (14) is linear, it suffices to prove the associated homogeneous system

(15) (a) (σ~h,∇vh)+(c·λ~h,vh)=0, ∀vh∈Vh,(b) (λ~h,wh)−(∇u~h,wh)=0, ∀wh∈Wh,(c) (σ~h,zh)+(aλ~h+bλ~ht,zh)=0, ∀zh∈Wh

has the trivial solution.

Choose $v_{h}={\tilde{u}}_{h}$ in (15)(a), $w_{h}={\tilde{\sigma }}_{h}$ in (15)(b), and $z_{h}={\tilde{\lambda }}_{h}$ in (15)(c) to obtain

(16) (a) (σ~h,∇u~h)+(c·λ~h,u~h)=0,(b) (λ~h,σ~h)−(∇u~h,σ~h)=0,(c) (σ~h,λ~h)+(aλ~h+bλ~ht,λ~h)=0.

Add the three equations to get

(17) ||a1/2λ~h||2+12ddt||b1/2λ~h||2  =12(btλ~h,λ~h)+(c·λ~h,u~h).

Integrate (17) with respect to time from 0 to *t* and use Cauchy-Schwarz inequality and Young inequality to obtain

(18) 2a0∫0t||λ~h||2ds+b0||λ~h||2  ≤C∫0t(||λ~h||2+||u~h||2)ds.

Taking $w_{h}=\nabla {\tilde{u}}_{h}$ in (15) and using Poincaré inequality, we obtain

(19) $$\begin{array}{c} ||{\tilde{u}}_{h}||\leq C||\nabla {\tilde{u}}_{h}||\leq C||{\tilde{\lambda }}_{h}||. \end{array}$$

Substitute (19) into (18) and use Gronwall lemma to obtain

(20) $$\begin{array}{c} \int_{0}^{t}{||{\tilde{\lambda }}_{h}||}^{2}ds+{||{\tilde{\lambda }}_{h}||}^{2}=0. \end{array}$$

From (20), we have

(21) $$\begin{array}{c} {\tilde{\lambda }}_{h}=0. \end{array}$$

Combining (19), (21), and (15)(c), we get

(22) $$\begin{array}{c} {\tilde{u}}_{h}=0,\;\;{\tilde{\sigma }}_{h}=0. \end{array}$$

Using (21) and (22), we get

(23) $$\begin{array}{c} {\tilde{u}}_{h}=0,\;\;{\tilde{\sigma }}_{h}=0,\;\;{\tilde{\lambda }}_{h}=0. \end{array}$$

In the following discussion, we will give some important lemmas based on new mixed scheme.

**Lemma 4**

There exists a linear operator Π_*h*_ : (*L* ^2^(*Ω*))^2^ → **W**_*h*_ such that

(24) $$\begin{array}{c} \left(\sigma -\Pi_{h}\sigma ,\nabla v_{h}\right)=0,\;\forall v_{h}\in V_{h}, \end{array}$$

(25) $$\begin{array}{c} {||\sigma -\Pi_{h}\sigma ||}_{L^{2}(\Omega )}\leq Ch{||\sigma ||}_{(H^{1}{\left(\Omega )\right)}^{2}}. \end{array}$$

**Lemma 5**

For the linear operator Π_*h*_ of Lemma 4, one has

(26) $$\begin{array}{c} \left(\lambda -\Pi_{h}\lambda ,w_{h}\right)=0,\;\forall w_{h}\in W_{h},\\ {||\lambda -\Pi_{h}\lambda ||}_{L^{2}(\Omega )}\leq Ch{||\lambda ||}_{(H^{1}{\left(\Omega )\right)}^{2}},\\ {||\lambda_{t}-\Pi_{h}\lambda_{t}||}_{L^{2}(\Omega )}\leq Ch{||\lambda_{t}||}_{(H^{1}{\left(\Omega )\right)}^{2}}. \end{array}$$

**Lemma 6**

There exists a linear operator *P*_*h*_ : *H*_0_^1^(*Ω*) → *V*_*h*_ such that

(27) $$\begin{array}{c} \left(\nabla \left(u-P_{h}u\right),w_{h}\right)=0,\;\forall w_{h}\in W_{h},\\ {||u-P_{h}u||}_{L^{2}(\Omega )}+h{||u-P_{h}u||}_{H^{1}}\leq Ch^{2}{||u||}_{H^{2}},\\ {||u_{t}-P_{h}u_{t}||}_{L^{2}(\Omega )}\leq Ch^{2}{||u_{t}||}_{H^{2}}. \end{array}$$

From [20, 21], we can obtain the proof for Lemmas 4–6.

Using the definition of Π_*h*_ and *P*_*h*_, we rewrite *η*, *δ*, and *ρ* as

(28) $$\begin{array}{c} \eta =u-{\tilde{u}}_{h}=u-P_{h}u+P_{h}u-{\tilde{u}}_{h}=\eta_{m}+\eta_{e};\\ \delta =\lambda -{\tilde{\lambda }}_{h}=\lambda -\Pi_{h}\lambda +\Pi_{h}\lambda -{\tilde{\lambda }}_{h}=\delta_{m}+\delta_{e};\\ \rho =\sigma -{\tilde{\sigma }}_{h}=\sigma -\Pi_{h}\sigma +\Pi_{h}\sigma -{\tilde{\sigma }}_{h}=\rho_{m}+\rho_{e}. \end{array}$$

Since estimates of *η*_*m*_, *δ*_*m*_, and *ρ*_*m*_ are known, it is enough to estimate *η*_*e*_, *δ*_*e*_, and *ρ*_*e*_. Using Lemmas 4–6, we rewrite (14) as

(29) (a)(ρe,∇vh)+(c·δe,vh)=0, ∀vh∈Vh,(b)(δe,wh)−(∇ηe,wh)=0, ∀wh∈Wh,(c)(ρe,zh)+(aδe,zh)+(b(δe)t,zh)  =−(aδm,zh)−(b(δm)t,zh), ∀zh∈Wh.

We discuss the following approximation properties for system (29).

**Lemma 7**

There is a constant *C* independent of *h* such that

(30) ||δ||≤Ch(||λ||(H1(Ω))2+∫0t(||u||H2+||λ||(H1(Ω))2+||λt||(H1(Ω))2)ds),

(31) ||δt||≤Ch(||u||H2+||λ||(H1(Ω))2+||λt||(H1(Ω))2+∫0t(||u||H2+||λ||(H1(Ω))2+||λt||(H1(Ω))2)ds),

(32) ||ρ||≤Ch(||u||H2+||σ||(H1(Ω))2+||λ||(H1(Ω))2+||λt||(H1(Ω))2+∫0t(||u||H2+||λ||(H1(Ω))2+||λt||(H1(Ω))2)ds),

(33) ||∇η||≤Ch(||u||H2+∫0t(||u||H2+||λ||(H1(Ω))2+||λt||(H1(Ω))2)ds).

**Proof**

Choose *v*_*h*_ = *η*_*e*_ in (29)(a), **w**_*h*_ = *ρ*_*e*_ in (29)(b), and **z**_*h*_ = *δ*_*e*_ in (29)(c) to obtain

(34) (a) (ρe,∇ηe)+(c·δe,ηe)=0,(b) (δe,ρe)−(∇ηe,ρe)=0,(c) (ρe,δe)+(aδe,δe)+(b(δe)t,δe)  =−(aδm,δe)−(b(δm)t,δe).

Add the three equations and use Cauchy-Schwarz inequality to get

(35) ||a1/2δe||2+12ddt||b1/2δe||2  =(c·δe,ηe)+12(btδe,δe)−(aδm,δe)−(b(δm)t,δe)  ≤C(||ηe||2+||δe||2+||δm||2+||(δm)t||2).

Integrate (35) with respect to time from 0 to *t* to obtain

(36) ||δe||2+∫0t||δe||2ds  ≤C∫0t(||ηe||2+||δe||2+||δm||2+||(δm)t||2)ds.

Using the Gronwall lemma, we have

(37) ||δe||2+∫0t||δe||2ds  ≤C∫0t(||ηe||2+||δm||2+||(δm)t||2)ds.

Differentiating (34)(b) and taking **w**_*h*_ = *ρ*_*e*_, we obtain

(38) $$\begin{array}{c} \left({\left(\delta_{e}\right)}_{t},\rho_{e}\right)-\left(\nabla {\left(\eta_{e}\right)}_{t},\rho_{e}\right)=0. \end{array}$$

Choose *v*_*h*_ = (*η*_*e*_)_*t*_ in (34)(a) and **z**_*h*_ = (*δ*_*e*_)_*t*_ in (34)(c) to obtain

(39) (a) (ρe,∇(ηe)t)=−(c·δe,(ηe)t),(c) (ρe,(δe)t)+||b1/2(δe)t||2  =−(aδe,(δe)t)−(aδm,(δe)t)−(b(δm)t,(δe)t).

Combining (38) and (39), we have

(40) ||b1/2(δe)t||2=−(c·δe,(ηe)t)−(aδe,(δe)t)−(aδm,(δe)t) −(b(δm)t,(δe)t)≤C(||ηe||2+||δe||2+||δm||2+||(δm)t||2)+b02||(δe)t||2.

Substitute (37) into (40) to obtain

(41) ||(δe)t||2≤C(||ηe||2+||δm||2+||(δm)t||2) +C∫0t(||ηe||2+||δm||2+||(δm)t||2)ds.

Choose **z**_*h*_ = *ρ*_*e*_ in (29)(c) and use Cauchy-Schwarz inequality (41) to obtain

(42) ||ρe||≤C(||ηe||2+||δm||2+||(δm)t||2) +C∫0t(||ηe||2+||δm||2+||(δm)t||2)ds.

Choose **w**_*h*_ = ∇*η*_*e*_ in (29)(b) and use (37) and Cauchy-Schwarz inequality to obtain

(43) $$\begin{array}{c} ||\nabla \eta_{e}||\leq C\int_{0}^{t}\left({||\eta_{e}||}^{2}+{||\delta_{m}||}^{2}+{||{\left(\delta_{m}\right)}_{t}||}^{2}\right)ds. \end{array}$$

Combining (37), (42), (43), and Lemmas 4–6 and using the triangle inequality, we get the conclusion of Lemma 7.

**Lemma 8**

There is a constant *C* independent of *h* such that

(44) ||η||≤Ch2||u||H2,||ηt||≤Ch2||ut||H2,||η||1≤Ch(||u||H2+∫0t(||u||H2+||λ||(H1(Ω))2+||λt||(H1(Ω))2)ds).

**Proof**

To estimate terms ||*η*||, ||*η*_*t*_||, and ||*η*||_1_, we consider the following auxiliary elliptic problem:

(45) $$\begin{array}{c} -\nabla \cdot \left(a\nabla \chi \right)=\eta ,\;\text{in}\;\;\Omega ,\\ \chi =0,\;\mathrm{on}\;\;\partial \Omega . \end{array}$$

Use (45) and Lemmas 4–6 to obtain

(46) ||η||2=(u−u~h,η)=(u−Phu+Phu−u~h,η)=(u−Phu,η)+(Phu−u~h,η)=(u−Phu,η)+(Phu−u~h,−∇·(a∇χ))=(u−Phu,η)+(∇(Phu−u~h),a∇χ)=(u−Phu,η)+(∇(Phu−u~h),a∇χ−Πh(a∇χ)) +(∇(Phu−u~h),Πh(a∇χ))=(u−Phu,η)≤||u−Phu||||η||≤Ch2||u||H2||η||.

From (46), we obtain

(47) $$\begin{array}{c} ||\eta ||\leq Ch^{2}{||u||}_{H^{2}}. \end{array}$$

Using similar method to ||*η*||_*L*^2^_, we can obtain

(48) $$\begin{array}{c} ||\eta_{t}||\leq Ch^{2}{||u_{t}||}_{H^{2}}. \end{array}$$

Combining (33) and (47), we obtain

(49) ||η||1≤Ch(||u||H2+∫0t(||u||H2+||λ||(H1(Ω))2+||λt||(H1(Ω))2)ds).

Using (47)–(49), we obtain the conclusion of Lemma 8.

For a priori error estimates, we decompose the errors as

(50) $$\begin{array}{c} u-u_{h}=u-{\tilde{u}}_{h}+{\tilde{u}}_{h}-u_{h}=\eta +\varsigma ;\\ \lambda -\lambda_{h}=\lambda -{\tilde{\lambda }}_{h}+{\tilde{\lambda }}_{h}-\lambda_{h}=\delta +\theta ;\\ \sigma -\sigma_{h}=\sigma -{\tilde{\sigma }}_{h}+{\tilde{\sigma }}_{h}-\sigma_{h}=\rho +\xi . \end{array}$$

Using (5)-(6) and (14), we can get the error equations

(51) (a)(ςt,vh)+(c·θ,vh)−(ξ,∇vh)=−(ηt,vh), ∀vh∈Vh,(b)(θ,wh)−(∇ς,wh)=0, ∀wh∈Wh,(c)(ξ,zh)+(aθ+bθt,zh)=0, ∀zh∈Wh.

We will prove the error estimates for semidiscrete scheme.

**Theorem 9**

Assume that $u_{h}(0)={\tilde{u}}_{h}(0)$ and $\lambda_{h}(0)={\tilde{\lambda }}_{h}(0)$; then one has the following estimates:

(52) $$\begin{array}{c} ||u-u_{h}||\leq Ch^{2}\left({||u||}_{H^{2}}+\int_{0}^{t}{||u_{t}||}_{H^{2}}ds\right),\\ ||u_{t}-u_{ht}||\leq Ch^{2}\left({||u_{t}||}_{H^{2}}+\int_{0}^{t}{||u_{t}||}_{H^{2}}ds\right),\\ {||u-u_{h}||}_{1}\leq Ch\left({||u||}_{H^{2}}+\int_{0}^{t}\left|||\cdot ||\right|ds\right),\\ ||\lambda -\lambda_{h}||\leq Ch\left({||\lambda ||}_{{\left(H^{1}(\Omega )\right)}^{2}}+\int_{0}^{t}\left|||\cdot ||\right|ds\right),\\ ||\lambda_{t}-\lambda_{ht}||\leq Ch\left({||\lambda_{t}||}_{(H^{1}{\left(\Omega )\right)}^{2}}+{||u_{t}||}_{H^{2}}+\int_{0}^{t}\left|||\cdot ||\right|ds\right),\\ ||\sigma -\sigma_{h}||\leq Ch\left({||\sigma ||}_{(H^{1}{\left(\Omega )\right)}^{2}}+{||u_{t}||}_{H^{2}}+\int_{0}^{t}\left|||\cdot ||\right|ds\right), \end{array}$$

where |||·|||≜||*u*||_*H*^2^_ + ||*u*_*t*_||_*H*^2^_ + ||*λ*||_(*H*^1^(*Ω*))^2^_ + ||*λ*_*t*_||_(*H*^1^(*Ω*))^2^_.

**Proof**

Choose *v*_*h*_ = *ς* in (51)(a), **w**_*h*_ = *ξ* in (51)(b), and **z**_*h*_ = *θ* in (51)(c) to obtain

(53) (a) 12ddt||ς||2−(ξ,∇ς)=−(ηt,ς)−(c·θ,ς),(b) (θ,ξ)−(∇ς,ξ)=0,(c) (ξ,θ)+||a1/2θ||2+12ddt||b1/2θ||2=12(btθ,θ).

Adding the above three equations, and using Cauchy-Schwarz inequality and Young inequality, we have

(54) 12ddt||ς||2+||a1/2θ||2+12ddt||b1/2θ||2  =−(ηt,ς)−(c·θ,ς)+12(btθ,θ)  ≤C(||ηt||2+||ς||2+||θ||2).

Integrate with respect to time from 0 to *t* to obtain

(55) ||ς||2+∫0t||θ||2ds+||θ||2  ≤C∫0t(||ηt||2+||ς||2+||θ||2)ds.

Using Gronwall lemma, we obtain

(56) $$\begin{array}{c} {||\varsigma ||}^{2}+\int_{0}^{t}{||\theta ||}^{2}ds+{||\theta ||}^{2}\leq C\int_{0}^{t}{||\eta_{t}||}^{2}ds. \end{array}$$

Take **w**_*h*_ = ∇*ς* in (51)(b) to have

(57) $$\begin{array}{c} ||\nabla \varsigma ||\leq ||\theta ||. \end{array}$$

Differentiating (51)(b) and taking **w**_*h*_ = *ξ*, we obtain

(58) $$\begin{array}{c} \;\left(\theta_{t},\xi \right)-\left(\nabla \varsigma_{t},\xi \right)=0. \end{array}$$

Choose *v*_*h*_ = *ς*_*t*_ in (51)(a) and **z**_*h*_ = *θ*_*t*_ in (51)(c) to obtain

(59) (a) ||ςt||2−(ξ,∇ςt)=−(ηt,ςt)−(c·θ,ςt),(c) (ξ,θt)+||b1/2θt||2=−(aθ,θt).

Adding (58), (59)(a), and (59)(c) and using Cauchy-Schwarz inequality, Young inequality, and (56), we have

(60) ||ςt||2+||b1/2θt||2=−(ηt,ςt)−(c·θ,ςt)−(aθ,θt)≤C||ηt||2+12||ςt||2+b02||θt||2 +C∫0t||ηt||2ds.

So, we have

(61) $$\begin{array}{c} {||\varsigma_{t}||}^{2}+{||\theta_{t}||}^{2}\leq C\left({||\eta_{t}||}^{2}+\int_{0}^{t}{||\eta_{t}||}^{2}ds\right). \end{array}$$

Choosing **z**_*h*_ = *ξ* in (51)(c) and using (56) and (61), we have

(62) 12||ξ||2=−(aθ+bθt,ξ)−12||ξ||2≤C(||θ||2+||θt||2)≤C(||ηt||2+∫0t||ηt||2ds).

Combining Lemmas 7 and 8, (56), (57), (61), (62), and the triangle inequality, we obtain the error estimate for Theorem 9.

## 4. Fully Discrete Scheme and Error Estimates

In this section, we get the error estimates of fully discrete schemes. For the backward Euler procedure, let 0 = *t*_0_ < *t*_1_ < *t*_2_ < ⋯<*t*_*M*_ = *T* be a given partition of the time interval [0, *T*] with step length Δ*t* = *T*/*M* and nodes *t*_*n*_ = *n*Δ*t*, for some positive integer *M*. For a smooth function *ϕ* on [0, *T*], define *ϕ* ^*n*^ = *ϕ*(*t*_*n*_) and ${\bar{\partial }}_{t}\phi^{n}=(\phi^{n}-\phi^{n-1})/\Delta t$.

Equation (5) has the following equivalent formulation:

(63) (a) (∂tun,v)+(cn·λn,v)−(σn,∇v)=(fn+R1n,v),∀v∈H01,(b) (λn,w)−(∇un,w)=0, ∀w∈(L2(Ω))2,(c) (σn,z)+(anλn+bn∂tλn,z)=(bnR2n,z),∀z∈(L2(Ω))2,

where

(64) $$\begin{array}{c} R_{1}^{n}=\partial_{t}u^{n}-u_{t}\left(t_{n}\right)=\frac{1}{\Delta t}\int_{t_{n-1}}^{t_{n}}‍\left(t_{n-1}-s\right)u_{tt}ds,\\ R_{2}^{n}=\partial_{t}\lambda^{n}-\lambda_{t}\left(t_{n}\right)=\frac{1}{\Delta t}\int_{t_{n-1}}^{t_{n}}\left(t_{n-1}-s\right)\lambda_{tt}ds.‍ \end{array}$$

Now we formulate a completely discrete procedure. Find (*u*_*h*_^*n*^, *λ*_*h*_^*n*^, *σ*_*h*_^*n*^) ∈ *V*_*h*_ × **W**_*h*_ × **W**_*h*_, (*n* = 0,1,…, *M*) such that

(65) (a) (∂tuhn,vh)+(cn·λhn,vh)−(σhn,∇vh)=(fn,vh),∀vh∈Vh,(b) (λhn,wh)−(∇uhn,wh)=0, ∀wh∈Wh,(c) (σhn,zh)+(anλhn+bn∂tλhn,zh)=0,∀zh∈Wh.

For the fully discrete error estimates, we now split the errors

(66) $$\begin{array}{c} u\left(t_{n}\right)-u_{h}^{n}=u\left(t_{n}\right)-{\tilde{u}}_{h}^{n}+{\tilde{u}}_{h}^{n}-u_{h}^{n}=\eta^{n}+\varsigma^{n};\\ \lambda \left(t_{n}\right)-\lambda_{h}^{n}=\lambda \left(t_{n}\right)-{\tilde{\lambda }}_{h}^{n}+{\tilde{\lambda }}_{h}^{n}-\lambda_{h}^{n}=\delta^{n}+\theta^{n};\\ \sigma \left(t_{n}\right)-\sigma_{h}^{n}=\sigma \left(t_{n}\right)-{\tilde{\sigma }}_{h}^{n}+{\tilde{\sigma }}_{h}^{n}-\sigma_{h}^{n}=\rho^{n}+\xi^{n}. \end{array}$$

We will prove the theorem for the fully discrete error estimates.

**Theorem 10**

Assume that $u_{h}^{0}={\tilde{u}}_{h}(0)$ and $\lambda_{h}^{0}={\tilde{\lambda }}_{h}(0)$; then there exists a positive constant *C* independent of *h* and Δ*t* such that

(67) ||u(tJ)−uhJ||≤Ch2(||u||2+||ut||2+||u||L∞(H2) +||ut||L∞(H2)) +CΔt∫t0tJ(||utt||+||λtt||)ds,||u(tJ)−uhJ||1≤Ch(||u||2+||u||L∞(H2)+||ut||L∞(H2)) +CΔt∫t0tJ(||utt||+||λtt||)ds,||λ(tJ)−λhJ||+a01/2(Δt∑n=1J||λ(tn)−λhn||2)1/2 ≤Ch(||λ||1+||σ||1+||u||L∞(H2)+||ut||L∞(H2))  +CΔt∫t0tJ(||utt||+||λtt||)ds,||σn−σhn||≤Ch(||λ||1+||σ||1+||u||2+||ut||2+||u||L∞(H2)+||ut||L∞(H2)) +Δt(||utt||L∞(L2)+||λtt||L∞(L2)) +CΔt∫t0tJ(||utt||+||λtt||)ds.

**Proof**

Using (14), (63), and (65) at *t* = *t*_*n*_, we get the error equations

(68) (a) (∂tςn,vh)+(cn·θn,vh)−(ξn,∇vh)  =−(∂tηn,vh)+(R1n,vh), ∀vh∈Vh,(b) (θn,wh)−(∇ςn,wh)=0, ∀wh∈Wh,(c) (ξn,zh)+(anθn+bn∂tθn,zh)=(bnR2n,zh), ∀z∈Wh.

Choose *v*_*h*_ = *ς* ^*n*^ in (68)(a), **w**_*h*_ = *ξ* ^*n*^ in (68)(b), and **z**_*h*_ = *θ* ^*n*^ in (68)(c) to obtain

(69) (a) 12Δt(||ςn||2−||ςn−1||2+||ςn−ςn−1||2)   +(cn·θn,ςn)−(ξn,∇ςn)=−(∂tηn,ςn)+(R1n,ςn),(b) (θn,ξn)−(∇ςn,ξn)=0,(c) (ξn,θn)+||(an)1/2θn||2   +12Δt(||(bn)1/2θn||2−||(bn−1)1/2θn−1||2+||(bn)1/2(θn−θn−1)||2)  =(bnR2n,θn)+(bn−bn−12Δtθn−1,θn−1).

Adding the above three equations, we obtain

(70) 12Δt(||ςn||2−||ςn−1||2+||(bn)1/2θn||2 −||(bn−1)1/2θn−1||2)+||(an)1/2θn||2 ≤−(∂tηn,ςn)+(R1n,ςn)−(cn·θn,ςn)  +(bnR2n,θn) ≤C(||∂tηn||2+||R1n||2+||R2n||2)  +C(||θn||2+||θn−1||2+||ςn||2).

Multiplying by 2Δ*t* and summing (70) from *n* = 1 to *J*, the resulting equation becomes

(71) (1−CΔt)||ςJ||2+(b0−CΔt)||θJ||2   +a0Δt∑n=1J||θn||2≤||ς0||2+b0||θ0||2   +CΔt∑n=1J(||∂tηn||2+||R1n||2+||R2n||2)   +CΔt∑n=1J−1(||θn||2+||ςn||2).

Choose Δ*t*_0_ in such a way that for 0 < Δ*t* ≤ Δ*t*_0_, (1 − *C*Δ*t*), (*b*_0_ − *C*Δ*t*) > 0. Then we use Cronwall's lemma to obtain

(72) ||ςJ||2+||θJ||2+a0Δt∑n=1J||θn||2  ≤CΔt∑n=1J(||∂tηn||2+||R1n||2+||R2n||2).

Note that

(73) $$\begin{array}{c} {||R_{1}^{n}||}^{2}\leq C\Delta t\int_{t_{n-1}}^{t_{n}}{||u_{tt}||}^{2}ds,\\ {||R_{2}^{n}||}^{2}\leq C\Delta t\int_{t_{n-1}}^{t_{n}}{||\lambda_{tt}||}^{2}ds,‍\\ {||\partial_{t}\eta^{n}||}^{2}\leq C\frac{1}{\Delta t}\int_{t_{n-1}}^{t_{n}}‍{||\eta_{t}(s)||}^{2}ds. \end{array}$$

Substitute (73) to (72) to get

(74) ||ςJ||2+||θJ||2+a0Δt∑n=1J||θn||2  ≤C∫t0tJ||ηt(s)||2ds   +C(Δt)2∫t0tJ(||utt||2+||λtt||2)ds.

Taking **w**_*h*_ = ∇*ς* ^*n*^ in (68)(b), we have

(75) $$\begin{array}{c} ||\nabla \varsigma^{n}||\leq ||\theta^{n}||. \end{array}$$

From (68)(b), we get

(76) $$\begin{array}{c} \left(\partial_{t}\theta^{n},w_{h}\right)-\left(\nabla \partial_{t}\varsigma^{n},w_{h}\right)=0. \end{array}$$

Choose **w**_*h*_ = *ξ* ^*n*^ in (76), *v*_*h*_ = ∂_*t*_ *ς* ^*n*^ in (68)(a), and **z**_*h*_ = ∂_*t*_ *θ* ^*n*^ in (68)(c) to obtain

(77) (a) ||∂tςn||2+(cn·θn,∂tςn)−(ξn,∇∂tςn)  =−(∂tηn,∂tςn)+(R1n,∂tςn),(b) (∂tθn,ξn)−(∇∂tςn,ξn)=0,(c) (ξn,∂tθn)+||(bn)1/2∂tθn||2  =−((an)1/2θn,∂tθn)+(bnR2n,∂tθn).

Adding the three equations, we obtain

(78) ||∂tςn||2+||(an)1/2∂tθn||2  ≤−(∂tηn,∂tςn)+(R1n,∂tςn)−(cn·θn,∂tςn)   +(bnR2n,∂tθn)−((an)1/2θn,∂tθn)  ≤C(||∂tηn||2+||R1n||2+||R2n||2)   +a02||∂tθn||2+12||∂tςn||2+C||θn||2.

Using (78) and (74), we get

(79) (||∂tςn||2+a0||∂tθn||2)  ≤Δt∑n=0J(||∂tηn||2+||R1n||2+||R2n||2)  ≤C(||ηt||L∞(L2)2+(Δt)2(||utt||L∞(L2)2+||λtt||L∞(L2)2))   +C∫t0tJ||ηt(s)||2ds   +C(Δt)2∫t0tJ(||utt||2+||λtt||2)ds.

Taking **z**_*h*_ = *ξ* ^*n*^ in (68)(c), we get

(80) ||ξn||2=−(anθn+bn∂tθn,ξn)≤C(||θn||2+||∂tθn||2)+12||ξn||2.

Substitute (79) into (80) to get

(81) ||ξn||2≤C(||ηt||L∞(L2)2+(Δt)2(||utt||L∞(L2)2+||λtt||L∞(L2)2)) +C∫t0tJ||ηt(s)||2ds +C(Δt)2∫t0tJ(||utt||2+||λtt||2)ds.

Combining (33)-(24), (74), (75), (81), and the triangle inequality, we complete the proof.

## 5. Numerical Example

In order to illustrate the efficiency of the new expanded mixed element method, we consider the following initial-boundary value problem of 2D Sobolev equation with the convection term:

(82) ut+c(x)·∇u−∇·(a(x,t)∇u+b(x,t)∇ut)=f(x,t),(x,t)∈Ω×J,u(x,t)=0, (x,t)∈∂Ω×J¯,u(x,0)=x1(x1−1)x2(x2−1), x=(x1,x2)∈Ω,

where *Ω* = [0,1]×[0,1], *J* = (0,1], *a*(**x**, *t*) = 1 + 2*x*_1_^2^ + *x*_2_^2^, *b*(**x**, *t*) = 1 + *x*_1_^2^ + 2*x*_2_^2^, and **c**(**x**) = (5,5)^*T*^, and *f*(**x**, *t*) is chosen so that the exact solution for the scalar unknown function is

(83) $$\begin{array}{c} u\left(x,t\right)=e^{-t}x_{1}\left(x_{1}-1\right)x_{2}\left(x_{2}-1\right), \end{array}$$

the corresponding exact gradient function is

(84) λ=∇u=(e−t(2x1−1)x2(x2−1),e−t(2x2−1)x1(x1−1)),

and its exact flux function is

(85) σ=−(a∇u+b∇ut)=(e−t(x22−x12)(2x1−1)x2(x2−1),e−t(x22−x12)(2x2−1)x1(x1−1)).

**Figure 1:**
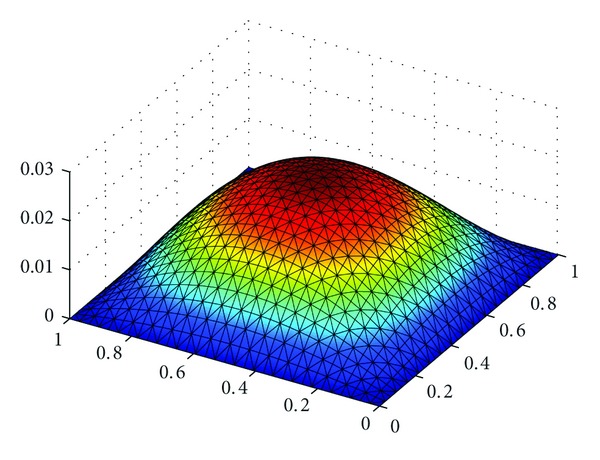
Surface for exact solution *u*.

**Figure 2:**
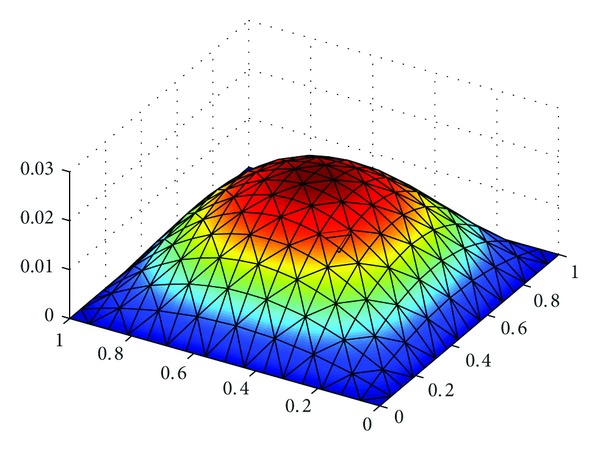
Surface for numerical solution *u*_*h*_.

**Figure 3:**
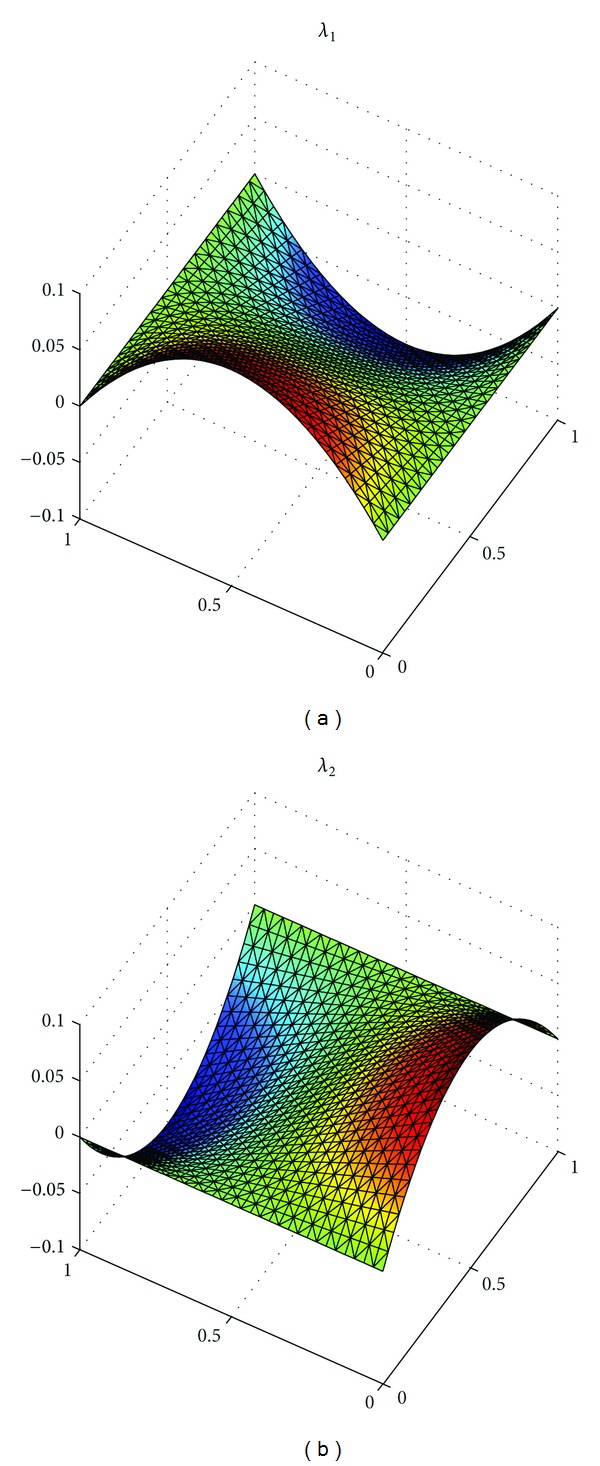
Surface for exact solution *λ* = (*λ*_1_, *λ*_2_).

**Figure 4:**
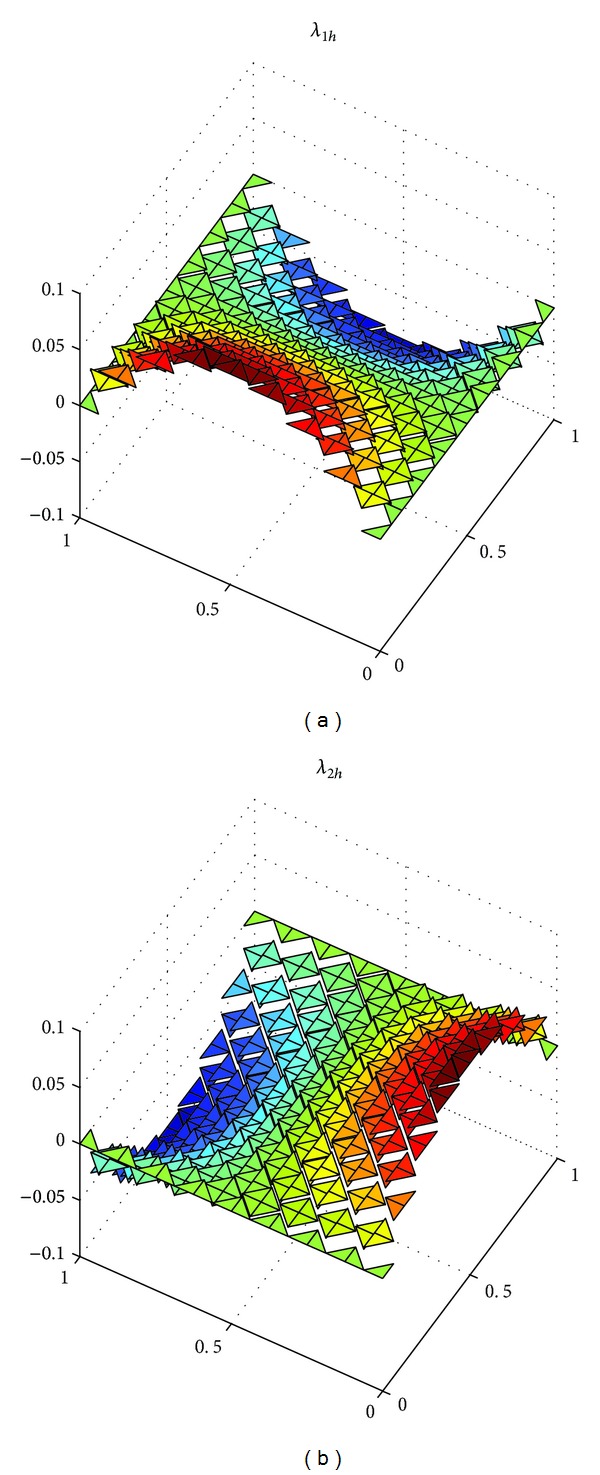
Surface for numerical solution *λ*_*h*_ = (*λ*_1*h*_, *λ*_2*h*_).

**Figure 5:**
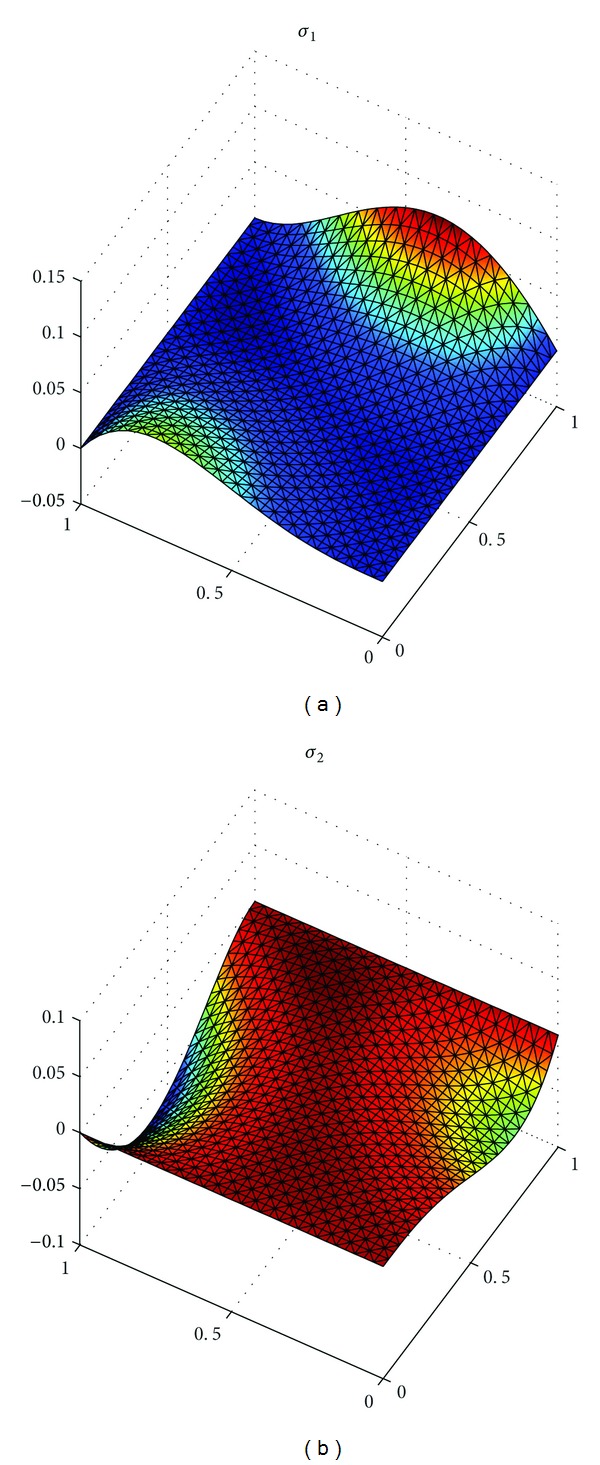
Surface for exact solution *σ* = (*σ*_1_, *σ*_2_).

**Figure 6:**
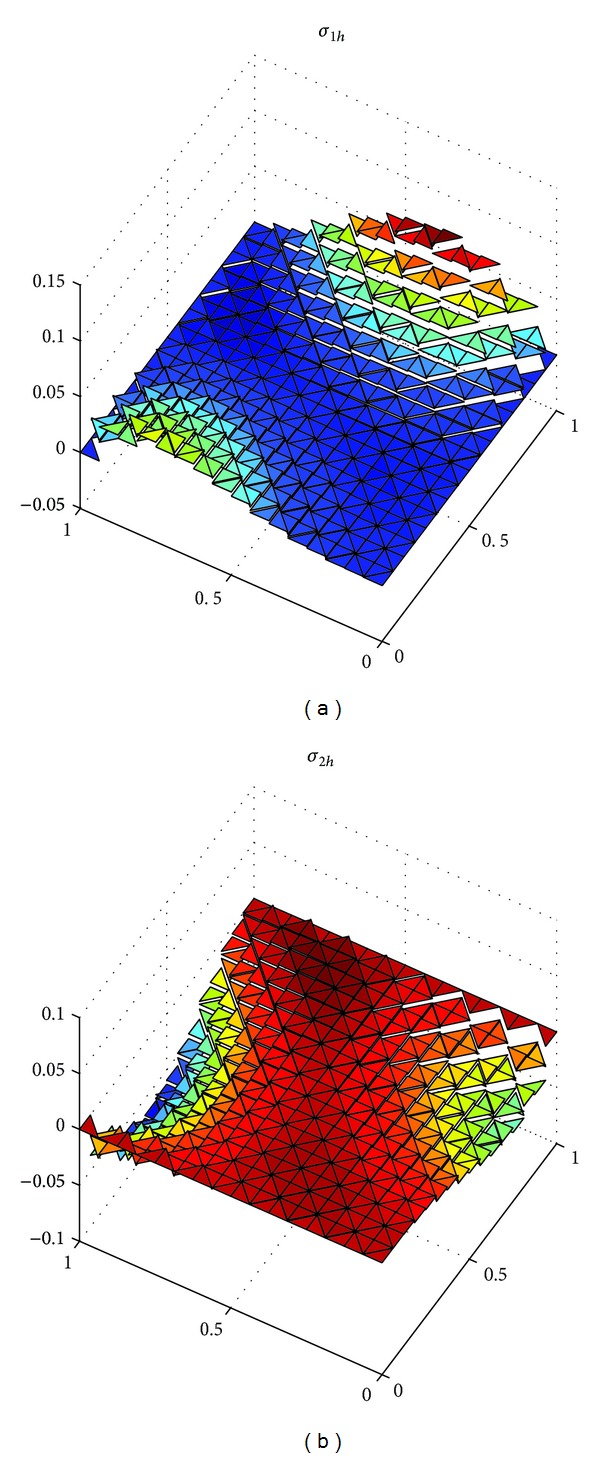
Surface for numerical solution *σ*_*h*_ = (*σ*_1*h*_, *σ*_2*h*_).

We divide the domain *Ω* into the triangulations of mesh size *h* uniformly, consider the piecewise linear space *V*_*h*_ with index *k* = 1 for the scalar unknown function *u* and the piecewise constant space **W**_*h*_ with index *k* = 0 for the gradient *λ* and the flux *σ*, use the backward Euler procedure with uniform time step length Δ*t* = 1/*M*, and obtain some convergence results for ||*u*−*u*_*h*_||_*L*^*∞*^(*L*^2^(*Ω*))_, ||*u*−*u*_*h*_||_*L*^*∞*^(*H*^1^(*Ω*))_, ||*λ*−*λ*_*h*_||_*L*^*∞*^((*L*^2^(*Ω*))^2^)_, and ||*σ*−*σ*_*h*_||_*L*^*∞*^((*L*^2^(*Ω*))^2^)_ with $h=2\sqrt{2}\Delta t=\sqrt{2}/8$, $\sqrt{2}/16$, $\sqrt{2}/32$ in Table 1. At the same time, we show the exact solutions *u*, *λ*, and*σ* in Figures 1, 3, and 5, respectively, and the corresponding numerical solutions *u*_*h*_, *λ*_*h*_, and *σ*_*h*_ in Figures 2, 4, and 6, respectively, with *t* = 1, and $h=2\sqrt{2}\Delta t=\sqrt{2}/16$.

It is easy to see that we obtained the optimal error estimates for *u* in *L* ^2^-norm, *H* ^1^-norm, and the error estimates for *λ* and *σ* in (*L* ^2^)^2^-norm, which confirm the theoretical results in this paper, in Table 1. The numerical results in Table 1 and Figures 1–6 show that new expanded mixed scheme for 2D Sobolev equation with convection term is efficient.

## 6. Concluding Remarks

In this paper, a new expanded mixed finite element method is proposed and studied for Sobolev equation with convection-term. The proof for the existence and uniqueness of the solution for semidiscrete scheme, the new expanded mixed projection, and the proof of its uniqueness are given. The optimal a priori error estimates in *L* ^2^ for the scalar unknown *u* and the a priori error estimates in (*L* ^2^)^2^-norm for its gradient *λ* and its flux *σ* are proved. Especially, the optimal a priori error estimates in *H* ^1^-norm for the scalar unknown *u* are derived. Finally, some numerical results are provided to confirm our theoretical analysis.

In the near future, the new expanded mixed method will be applied to other evolution equations such as evolution integrodifferential equations, hyperbolic wave equations, and nonlinear evolution equations. And the new characteristic expanded mixed finite element method for Sobolev equation will be studied. The new expanded characteristic-mixed weak formulation is to find {*u*, *λ*, *σ*}:[0, *T*] ↦ *H*_0_^1^ × (*L* ^2^(*Ω*))^2^ × (*L* ^2^(*Ω*))^2^ such that

(86) (a) (ψ∂u∂τ,v)−(σ,∇v)=(f,v), ∀v∈H01,(b) (λ,w)−(∇u,w)=0, ∀w∈(L2(Ω))2,(c) (σ,z)+(aλ+bλt,z)=0, ∀z∈(L2(Ω))2,

where

(87) $$\begin{array}{c} \psi \left(x,t\right)={\left(1+{\left|c(x)\right|}^{2}\right)}^{1/2},\\ \frac{\partial }{\partial \tau \left(x\right)}=\frac{1}{\psi \left(x,t\right)}\frac{\partial }{\partial t}+\frac{c}{\psi \left(x,t\right)}\cdot \nabla . \end{array}$$

In another article, we will give the error estimates for the new characteristic expanded mixed finite element method.

## Figures and Tables

**Table 1:** Errors and order of convergence.

(*h*, Δ*t*)	||*u*−*u*_*h*_||_*L*^∞^(*L*^2^(Ω))_	Order	||*u*−*u*_*h*_||_*L*^∞^(*H*^1^(Ω))_	Order
$\left(\sqrt{2}/8,1/16\right)$	1.4059*e* − 003	—	1.8754*e* − 002	—
$\left(\sqrt{2}/16,1/32\right)$	3.6420*e* − 004	1.9486	9.6596*e* − 003	0.9572
$\left(\sqrt{2}/32,1/64\right)$	9.2569*e* − 005	1.9761	4.9011*e* − 003	0.9789

(*h*, Δ*t*)	||*λ*−*λ*_*h*_||_*L*^∞^((*L*^2^(Ω))^2^)_	Order	||*σ*−*σ*_*h*_||_*L*^∞^((*L*^2^(Ω))^2^)_	Order

$\left(\sqrt{2}/8,1/16\right)$	1.8701*e* − 002	—	9.7089*e* − 003	—
$\left(\sqrt{2}/16,1/32\right)$	9.6528*e* − 003	0.9541	5.1777*e* − 003	0.9070
$\left(\sqrt{2}/32,1/64\right)$	4.9003*e* − 003	0.9781	2.6722*e* − 003	0.9543
